# Secondary Traumatization Outcomes and Associated Factors Among the Health Care Workers Exposed to the COVID-19

**DOI:** 10.1177/0020764020940742

**Published:** 2020-07-08

**Authors:** Selim Arpacioglu, Meltem Gurler, Suleyman Cakiroglu

**Affiliations:** 1 Department of Child and Adolescent Psychiatry, Istanbul Medeniyet University, Goztepe Education and Research Hospital, Istanbul, Turkey; 2 Clinical Psychology Graduate Student, Istanbul Kent University, Istanbul, Turkey; 3 Department of Child and Adolescent Psychiatry, Istanbul Medeniyet University, Goztepe Education and Research Hospital, Istanbul, Turkey

**Keywords:** COVID-19, health care workers, secondary traumatization, depression, anxiety, risk factors

## Abstract

**Background:**

Secondary traumatization exposure and mental health conditions of health care workers gained importance during the coronavirus disease 2019 (COVID-19) epidemic period.

**Aims:**

In our study, we aim to research the secondary traumatization and associated factors among health care workers.

**Method:**

This cross-sectional study was applied through an online questionnaire using the snowball sampling method. Two hundred fifty-one health care workers from different units/services and 312 non-medical worker adults attended to the research. Health care workers were divided into two groups based on working with COVID-19 patients at the frontline or not. The data were collected via Introductory Information Form, Secondary Traumatic Stress Scale (STSS) and Patient Health Questionnaire-4 (PHQ-4) between 22 May and 30 May 2020.

**Results:**

Among the 563 participants, 251 (44.6%) were health care workers and 312 (55.4%) were non-medical workers. The anxiety, depression and secondary traumatization scores of the frontline health care workers for the COVID-19 were found to be significantly higher than those of the other health workers or non-medical workers (*p* < .001). Also, we found that being a woman, being in the first years of the work, living with a parent, having a chronic disease, having a trauma history and increased social media use are related to having higher scores from the secondary traumatization scale.

**Conclusion:**

The secondary traumatization exposure and the mental health conditions of the health care workers directly working with the COVID-19 patients should be taken into consideration. It is important to provide social support, examine and control riskier groups for mental health regularly during the pandemic.

## Introduction

The coronavirus disease 2019 (COVID-19) outbreak has currently affected more than 3.5 million people globally between the December 2019 and May 2020 period. The World Health Organization (WHO) has declared the outbreak a pandemic in March 2020 and stated that the world was facing a global health crisis (World Health Organization, 2020). Consequently, many countries around the world have implemented precautionary measures and quarantines to prevent the virus from spreading.

During the outbreak, the lack of any specific treatments or a vaccine, and factors like the hardships of lockdown (and social distancing) measures, not knowing when the measures will be loosened and bleak economic outlook have been determined to affect the mental health of individuals (Bao et al., 2020; Deng & Peng, 2020; Wang et al., 2020). A study conducted during the first severe acute respiratory syndrome (SARS) outbreak in 2003 found that patients being cared for in hospitals had experienced increasing levels of fear, loneliness, anger, sleep deprivation and anxiety (Maunder et al., 2003). Similarly, Shigemura et al. (2020) have remarked that the COVID-19-infected patients might be prone to developing stress reactions (sleep deprivation, anger, fear of sickness), risky behavior (increasing alcohol or tobacco consumption) and psychiatric disorders (depression, somatization, post-traumatic stress disorder (PTSD), anxiety, etc.).

Frontline health care workers against COVID-19 are considered particularly susceptible to developing psychiatric disorders (Lai et al., 2020). All health care workers, and particularly nurses in similar outbreaks, have suffered an increase in mental pressure due to the heavy workloads they have taken and lack of satisfactory personal protective equipment (PPE) and medication, as well as being exposed to a deathly virus for extended periods. In addition, having to stay away from their families has compounded this mental strain, as fear of infecting loved ones has posed another psychological burden to the health care workers (Lai et al., 2020; Xiang et al., 2020). During the 2003 SARS outbreak, health professionals suffered high levels of stress and anxiety and showed depression symptoms (Maunder et al., 2003). However, there are currently limited data about the effects of the COVID-19 pandemic on health care workers in literature. According to a study conducted on 1,830 frontline health care workers in China, approximately half of the respondents (50.4%) showed signs of depression, 44.6% of anxiety, 34% of insomnia and 71.5% have shown symptoms of increased stress (Lai et al., 2020). Likewise, a study carried out in two hospitals caring for COVID-19 patients in Singapore found that 14.5% of the 470 health care workers had shown symptoms of anxiety, whereas 6.6% had shown symptoms of depression (Tan et al., 2020). Lai et al. (2020) report has further confirmed that health professionals during the COVID-19 outbreak have reported higher levels of stress, sleep deprivation, depression and anxiety (Lai et al., 2020).

Moreover, it has been known that COVID-19 patients who are cared for in hospitals in the intensive care unit or isolation units are likely to experience shortness of breath and fear of death. These experiences can make a traumatic effect on them (Horesh & Brown, 2020; Li et al., 2020). Professionals who work for extended amounts of time with patients with previous traumas are similarly prone to experiencing secondary trauma (Collins & Long, 2003). It has been documented that these instances of secondary trauma can cause loss of appetite, sleep deprivation, fatigue, anger, apathy and hopelessness in the affected individuals (Creighton et al., 2018).

In consideration of the lack of previous research into secondary trauma in health care professionals, this article aims to study the differences in secondary traumas experienced by the frontline workers, by the broader category of health care professionals and finally by the general public. Our results aim to help in the early recognition and remedying of secondary trauma in health care professionals, as well as identifying the important risk factors for future research.

## Methods

### Study design and sample

This study was fulfilled in a cross-sectional and descriptive design. We used an online survey to minimize face-to-face interactions with all participants and to facilitate participation of health care workers who work extensively during this emergency period. A convenience sample of health care workers and the general population were contacted to participate in this study. The survey was shared on various social network groups from different health care professions to gather health care workers. Participants also asked to share the survey with their colleagues and with the health care workers they know. After collecting the data of health care workers, we shared the survey form at various social network groups and asked participants to share the survey with their friends or family who is older than 18 years and a non–health care worker. The sample of the study was determined by using the snowball sampling method. The respondents consist of 251 health care workers from different units and 312 non-medical worker adults older than 18 years. All the participants were from Turkey, and as we used snowball sampling and online surveys, we gathered subjects from several institutions. The Introductory Information Form, Secondary Traumatic Stress Scale (STSS) and Patient Health Questionnaire-4 (PHQ-4) were used as data collection tools. The data were collected online from 22 May to 30 May 2020. Before collecting the data, all respondents were informed about the aim of the study, data privacy and only scientific intended use of the data. An informed consent was received from all respondents. To be able to carry out this study, approvals from Kent University clinical research ethics committee (28.05.2020/2020-04, 77083609-100/136) and Ministry of Health were received.

### Data collection tools

#### Introductory Information Form

This form, designed by the researchers, asks demographic questions such as gender, age, educational background, marital status and number of children. The medical worker respondents were asked if they are directly associated with the diagnosis, treatment or nursing services of the COVID-19-positive patients, and the respondents who answered this question affirmatively were accepted as frontline workers and the respondents who answered negatively were accepted as background workers. The form revealed if the respondents had any traumatic life experiences (after giving a detailed description of trauma). Also, the respondents were asked overall assessment questions such as if they have someone close to them who have a disease or passed away due to the pandemic, how much time they spent on social media during the pandemic and if they have any psychological or medical diseases, and the subjective changes in their anxiety levels.

#### STSS

The STSS is a 17-item 5-point Likert-type self-report scale designed by Bride and colleagues (2004). The scale was developed to measure the secondary post-traumatic stress symptoms, and it evaluates individual’s reactions in the last 7 days. The obtained points from the scale vary between 17 and 85, and higher points indicate a higher affection level. Turkish reliability and validity of the scale was performed by Yıldırım and colleagues (2017; Bride et al., 2004).

#### PHQ-4

This four-question scale was designed by Kroenke and colleagues (2009), and it evaluates anxiety and depression symptoms. The first two questions examine anxiety, and the other two questions examine depression complaints. Each question gets scored between 0 and 3 depending on how often the respondents have bothered by the problems over the last 2 weeks. Turkish adaptation of the scale was performed by Demirci and Eksi (2018; Kroenke et al., 2009).

### Statistical analysis

Statistical analysis was performed by using the SPSS version 24.0 (IBM Corp., Armonk, NY, ABD) packaged software. Significance levels of all outcomes were taken as α = .05, and analyses were made two-way. The normality assumption of the data was evaluated with normality tests (Kolmogorov–Smirnov vs Shapiro–Wilk), and it showed normal distribution with a 95% trust. Sociodemographic attributes of the respondents were determined by using descriptive statistics (number, percentage, mean, standard deviation). The relation between the sociodemographic attributes and scale total and subdimension points was examined with the Pearson correlation analysis and one-way analysis of variance (ANOVA). The differences between the sociodemographic attributes and scale total and subdimension points were analyzed with a *t* test in independent groups. To specify the factors that affect the respondents’ secondary traumatization level, multivariate logistic regression analysis was performed, and the relations and rates between risk factors and results were evaluated considering a 95% confidence interval.

## Results

### Demographic attributes

Five hundred sixty-three participants, 212 (37.7%) men and 351 (62.3%) women aged between 20 and 61 years (*M*: 32 ± 10.2), completed the research. Three hundred seventy (65.7%) were married, 137 (24.3%) were single and 56 (9.9%) were divorced or widowed. Two hundred fifty-one (44.6%) were health care workers and 312 (55.4%) were from different occupations. One hundred twenty-four (49%) of the health care workers were doctors and 93 (37%) were nurses. Thirty-four (14%) of the health care workers were hospital personnel such as biochemistry technicians, physiotherapists and janitors. One hundred nine (43.4%) of the health care workers were directly working with the COVID-19-positive patients and 142 (56.6%) were not in direct contact. Three hundred sixty-two (64.3%) respondents lived with their spouses and children, 79 (14%) lived with their parents and 67 (11.9%) lived alone. The sociodemographic attributes of the respondents and their answers to the questions were represented in Table 1.

**Table 1.** Demographic attributes.

	*n*	%
Gender		
Female	351	62.3
Male	212	37.7
Marital status		
Single	137	24.3
Married	370	65.7
Divorced/widowed	56	9.9
How many children do you have?		
0	207	36.8
1	142	25.2
2	192	34.1
3 or more	22	3.9
Whom do you live with?		
Alone	67	11.9
My spouse or/and children	362	64.3
My parents	79	14.0
Other	55	9.8
Changes in general anxiety level after the pandemic		
Same	148	26.3
Increased	415	73.7
Occupations		
Not health care worker	312	55.4
Dentist	27	4.8
Doctor	97	17.2
Nurse	93	16.5
Other health care worker	34	6.0
How long have you been working in the health sector?		
0–4 years	46	18.3
5–9 years	40	15.9
10–19 years	80	31.9
20 years and more	85	33.9
Are you a governmental institution or a private firm health care worker?		
Government	88	35.1
Private/foundation	163	64.9
Do you work with the COVID-19-positive patients?		
Yes	109	19.4
No	142	25.2
Not health care worker	312	55.4
Have you ever experienced a traumatic event?		
Yes	227	40.3
No	336	59.7
How much time do you spend on social media (WhatsApp, Twitter, Instagram, etc.) after the pandemic?		
Less	48	8.5
Same	166	29.5
More	260	46.2
Much more	89	15.8

COVID-19: coronavirus disease 2019.

### Severity of measurements and differences between the groups

The demographic attributes of the respondents such as age, marital status, having a child and having other health care workers in the family were compared with their secondary traumatization scale points, and no significant difference was found (*p* < .05). Also, there is no significant difference between the health care worker respondents’ secondary traumatization scale total points and their institutions, conditions, occupational groups, units, weekly working hours and number of patients (*p* < .05).

The difference between the secondary traumatization scale and PHQ total point average of the respondents who directly work with the COVID-19-positive patients and the respondents who do not directly work with the COVID-19-positive patients was compared with one-way ANOVA, and a statistically significant difference was found (*p* < .001). While the health care worker group who work with the COVID-19-positive patients got the highest score, the non-medical worker group got the lowest score (Table 2).

**Table 2.** The Severity of measurements and differences between the groups.

Variables	*n*	Secondary trauma	Patient health scale
		Total *M* ± *SD*	Total *M* ± *SD*
Gender			
Female	351	2.53 ± 0.82	2.05 ± 0.69
Male	212	2.22 ± 0.82	1.79 ± 0.63
		*t*: 4.43 *p* < .001	*t*: 4.53 *p* < .001
Whom do you live with?			
Alone	67	2.24 ± 0.72	1.85 ± 0.60
My spouse and/or children	362	2.40 ± 0.82	1.86 ± 0.64
My parents	79	2.59 ± 0.97	2.36 ± 0.72
		*f*: 2.37 *p*: .069	*f*: 13.63 *p* < .001
Working with the COVID-19-positive patients			
Yes	109	2.66 ± 0.96	2.25 ± 0.79
No	142	2.46 ± 0.83	2.01 ± 0.70
Not a health care worker	312	2.34 ± 0.76	1.82 ± 0.59
		*f*: 6.25 *p* < .001	*f*: 17.73 *p* < .001
Social media use after the pandemic			
Less	48	2.37 ± 0.90	2.19 ± 0.90
Same	166	2.18 ± 0.78	1.83 ± 0.65
More	260	2.46 ± 0.78	1.92 ± 0.63
Much more	89	2.72 ± 0.93	2.15 ± 0.67
		*f*: 8.98 *p* < .001	*f*: 6.55 *p* < .001
Past trauma history			
Yes	227	2.61 ± 0.89	2.12 ± 0.73
No	336	2.27 ± 0.76	1.83 ± 0.61
		*t*: 4.88 *p* < .001	*t*: 4.97 *p* < .001
Chronic disease history			
No	343	2.34 ± 0.83	1.92 ± 0.68
Yes but not risky	155	2.48 ± 0.81	1.95 ± 0.62
Yes and risky	65	2.66 ± 0.87	2.12 ± 0.80
		*f*: 4.58 *p*: .011	*f*: 2.27 *p*: .104
Working years in the medical sector			
0–4	46	2.45 ± 0.91	2.36 ± 0.81
5–9	40	2.83 ± 0.92	2.46 ± 0.76
10–19	80	2.56 ± 0.090	2.16 ± 0.74
20 and more	85	2.31 ± 0.88	1.79 ± 0.57
		*f*: 3.29 *p*: .021	*f*: 11.22 *p* < .001

COVID-19: coronavirus disease 2019.

Besides, being a woman, being in the first years of the work, living with a parent, having chronic diseases, having a trauma history in the past and increased use of social media were found related to scoring significantly higher points from the secondary traumatization scale (Table 2).

### Risk factors of secondary traumatization

The effects of the variables on the secondary traumatization were analyzed with simple linear regression, and the output values were represented in Table 3. Analyses showed that gender, age, anxiety and depression levels, anxiety about the COVID-19 and past trauma history are independent risk factors for secondary traumatization (*p* < .05).

**Table 3.** Risk factors for secondary traumatization analyzed with simple linear regression.

Variables	*B*	*SE*	β	*p* value
Age	0.08	0.04	.07	.04
Gender	2.59	0.45	.26	.00
Patient health (anxiety)	2.80	0.41	.31	.00
Patient health (depression)	−2.03	0.97	−.07	.04
Anxiety about the COVID-19	6.87	1.14	.21	.00
Trauma history	2.22	0.95	.08	.02

COVID-19: coronavirus disease 2019.

## Discussion

Studies state that the COVID-19 pandemic can cause many psychological effects, such as anxiety, fear, depression and stress on people, especially on health care workers (Ahmed et al., 2020; Bao et al., 2020; Deng & Peng, 2020; Jiao et al., 2020; Lai et al., 2020; Shigemura et al., 2020). This study aims to evaluate the anxiety and depression signs of the health care workers exposed to secondary traumatization during the pandemic. Five hundred sixty-three participants, 251 of whom are health care professionals, attended this cross-sectional study, and we found that health care workers who treat the COVID-19 patients experience higher secondary traumatization. In this way, we can think that working as a frontline health care worker is an important risk factor for negative consequences on mental health. Our findings present a necessity for primary assessment of frontline health care workers’ mental health.

It was reported that frontline health care workers have a higher risk for mental health (Lai et al., 2020; Tan et al., 2020). Fear of being infected and infecting their loved ones, heavy workload, limited material and decrease in social support are the factors related to the anxiety and depression signs (Chua et al., 2004; Lai et al., 2020; Wong et al., 2005). Also, the rapid spread of the COVID-19 from person to person, being constantly exposed to the virus, high morbidity and potential death risk can cause secondary traumatization by intensifying the danger perception (Chen et al., 2020; Lai et al., 2020; Wang et al., 2020).

Also, in our study, we found that being a woman, being in the first years of the work, living with a parent, having a chronic disease, trauma history and increased use of social media are associated with high secondary traumatization and patient health scale points. Several studies suggest that female health care workers are affected by the pandemic more than others. When considered many female health care workers who treat the COVID-19 patients are nurses, they can carry a higher risk for mental health due to the close and frequent contact with the patients and longer working hours than usual (Chan, 2003; Chen et al., 2020; Lai et al., 2020; Zhang & Ma, 2020). Considering our findings, one can see that health care workers with 10 years or less of work experience have higher secondary traumatization points. Our study findings showed the group who was working for 20 years or more as health care worker has the least traumatization, depression and anxiety. In parallel with our research, Fried and Fisher’s study in 2016 asserted employees with less experience have a higher level of stress and burnout related to work. People who live with their parents have higher points of both secondary traumatization and anxiety, and it can be related to the severity and the fatality of COVID-19 disease among the elders. The health care workers with a traumatic history in their past have significantly high secondary traumatization points. Related studies presented that past traumas are risky for the future PTSD. During the pandemic, participants who used social media more than usual have significantly higher secondary traumatization points. Unfounded information and speculative news on social media, being frequently exposed to the pandemic-related news, witnessing the ambiguity of the further pandemic process and keeping track of the death toll can be counted as the reasons for high secondary traumatization points.

There are various limitations to this research. One of the limitations is that the research was conducted through an online questionnaire. It was applied cross-sectional and could not be pursued longitudinally due to the long working hours and social isolation rules of the health care workers. Another limitation is that the data collected only through an online questionnaire and a structured or semi-structured interview could not be actualized. So, the study does not have a strong sampling frame; therefore, the results may not be generalizable (Pierce et al., 2020). But we decided to do the study with the idea that it is important to document these data about the secondary traumatization that health care workers and general population experience during the COVID process.

## Conclusion

In Turkey, frontline health care workers who work with the COVID-19 patients stated a high level of secondary traumatization. High traumatization values were found related to being a woman, living with a parent, having less work experience, having a trauma history in the past and increased use of social media. In this case, considering the risk factors, protecting the health care workers and taking precautions are very important (Greenberg et al., 2020).
